# Strength Development and Hydration Behavior of Self-Activation of Commercial Ground Granulated Blast-Furnace Slag Mixed with Purified Water

**DOI:** 10.3390/ma9030185

**Published:** 2016-03-10

**Authors:** Hyeoneun Park, Yeonung Jeong, Jae-Hong Jeong, Jae Eun Oh

**Affiliations:** 1 School of Urban and Environmental Engineering, Ulsan National Institute of Science and Technology (UNIST), UNIST-gil 50, Ulju-gun, Ulsan 689-798, Korea; pphe1789@unist.ac.kr (H.P.); yjeong@unist.ac.kr (Y.J.); 2 Samsung C&T Corporation, 14, Seocho-daero 74-gil, Seocho-gu, Seoul 137-956, Korea; jackslo@samsung.com

**Keywords:** GGBFS, calcium sulfates, hydration with water, cementless binder, strength

## Abstract

In this study, ground granulated blast-furnace slag (GGBFS) samples from Singapore, Korea, and the United Arab Emirates were hydrated with purified water to estimate the cementing capabilities without activators. Raw GGBFS samples and hardened pastes were characterized to provide rational explanations for the strengths and hydration products. The slag characteristics that influenced the best strength of raw GGBFS were identified. Although it is widely recognized that GGBFS alone generally shows little cementing capability when hydrated with water, the GGBFSs examined in this study demonstrated various strength developments and hydration behaviors; one of the GGBFS samples even produced a high strength comparable to that of alkali- or Ca(OH)_2_-activated GGBFS. In particular, as the GGBFS exhibited a greater number of favorable slag characteristics for hydraulic reactivity, it produced more C-S-H and ettringite. The results demonstrated a reasonable potential for commercial GGBFS with calcium sulfates to function as an independent cementitious binder without activators.

## 1. Introduction

The American Concrete Institute (ACI) and the American Society for Testing and Materials (ASTM) previously used the terminology “ground granulated blast-furnace slag” (GGBFS) to denote the ground form of glassy granular material that develops when molten blast-furnace slag, which is an industrial byproduct waste of steel manufacturing, is rapidly cooled.

In 2003, however, “GGBFS” was replaced with the term “slag cement” by the ACI (ACI 233R-03) [1] and the ASTM (C125-11) [2], at the request of GGFBS manufacturers (the details of the request and discussion on the terminology are not provided in these articles). Thus, GGBFS, or slag cement, gradually came to be recognized as a separate cementitious constituent rather than as a supplementary material for concrete production. Despite the new terminology, the self-cementing ability of GGBFS mixed with pure water continues to be ignored in related academia and industry.

When GGBFS is hydrated with pure water (hereafter, this reaction is denoted as the “self-activation of GGBFS”), a protective film quickly forms on the surface of the GGBFS particles, resulting in a very slow hydration [3]; thus, it is widely recognized that GGBFS alone shows few cementing properties when mixed with pure water [4,5,6] unless they are activated with activating chemicals such as alkaline hydroxides, soluble silicates, Portland cement, or lime [4,5,6,7,8,9].

However, when calcium sulfates (e.g., CaSO_4_, and CaSO_4_·2H_2_O) are incorporated into GGBFS, the behavior of its self-activation could be significantly different from that of GGBFS without any calcium sulfates, because these chemicals may considerably promote the hydration of GGBFS with even small quantities of activators [10]; thus, the strength of the self-activated paste of commercial GGBFS could be much greater than expected.

Therefore, given that most commercial GGBFS powders in current markets contain additional calcium sulfates in the forms of anhydrite (CaSO_4_), hemihydrate (CaSO_4_·1/2H_2_O), or gypsum (CaSO_4_·H_2_O), from an engineering perspective, the self-activation of commercial GGBFS with calcium sulfates is more important than that of GGBFS without calcium sulfates.

Currently, however, no study to date has investigated the self-activation of commercial GGBFS, although a few earlier studies briefly introduced the self-activation of noncommercial GGBFS [4,5,6]. In this study, three commercial GGBFS samples were purchased from three different locations (*i.e*., Korea, Singapore, and the United Arab Emirates). The GGBFS samples were hydrated with deionized (DI) water and characterized using compressive strength testing, X-ray fluorescence (XRF), power X-ray diffraction (XRD), a laser diffraction particle size analyzer, and a thermogravimetric analyzer (TGA).

## 2. Experimental Program

This study investigated three GGBFS samples that were commercially available for construction in Singapore (labeled S-Slag), the Republic of Korea (labeled K-Slag), and Dubai, in the United Arab Emirates (labeled D-Slag). S-Slag, a commercial GGBFS, was ground with additives in Singapore, but the raw granulated blast-furnace slag (GBFS) was imported from Japan. Likewise, D-Slag was manufactured in Dubai, but its original GBFS was a blended material using GBFSs from China and India, while K-Slag was manufactured using raw GBFS from Korea. Each vendor used different types and quantities of additives in the GGBFS production.

The raw GGBFSs were examined using XRF (S8 Tiger wavelength dispersive (WDXRF) spectrometer, Bruker, Billerica, MA, USA), powder XRD (high-power X-ray diffractometer, Rigaku, Tokyo, Japan), and laser diffraction particle-size analysis (HELOS (HI 199) and RODOS, Sympatec, Clausthal-Zellerfeld, Germany) to determine basic material properties. Loss-of-ignition (LOI) values for the raw GGBFS samples were determined using TGA (SDT Q600, TA Instruments, New Castle, DE, USA).

The GGBFSs were mixed only with deionized (DI) water; each was homogenized by dry-stirring for five minutes and mixed with DI water at a weight ratio of water-to-GGBFS (w/G) of 0.4. All mixing procedures were conducted following ASTM C305 [11]. The freshly mixed pastes were cast in cubic molds of 5 cm × 5 cm × 5 cm for compressive strength testing. The strength result for each mixture was reported as an average value of the testing results of three identical specimens.

Small fractions of pastes were diluted with DI water at a weight ratio of water to GGBFS (w/G) = 2 to monitor the changes of the diluted pastes’ pH values for seven days to obtain approximate estimates of the pH of the pore solutions. For more accurate measurements, the pH of the pore solution should be measured directly after being extracted from the hardened paste; however, an earlier study [4] demonstrated that the pH value trends of the diluted pastes were not different from those of the extracted solutions in early days.

Following the compressive strength testing, the fractured inner pieces were collected and finely ground for the XRD and TGA. Each XRD test was performed on the same day that each compressive strength testing was conducted. The XRD patterns for the raw GGBFSs and the hardened pastes were recorded using a high-power powder X-ray diffractometer (Rigaku, Tokyo, Japan) with Cu-Kα radiation (λ = 1.5418 Å) within a range of 50°–60° in 2θ. An internal standard (NIST RMS 676a, crystalline alumina 99.02% ± 1.11%) was used to calculate the weight content of the glass phase of the raw GGBFS [12]. All XRD patterns were analyzed using X’pert HighScore Plus software [13] with the Internal Center for Diffraction Data (ICDD) PDF-2 database [14] and the Inorganic Crystal Structure Database (ICSD) [15].

The TGA data were collected using an SDT Q600 (TA Instruments, New Castle, DE, USA) with an alumina pan, from ambient temperature to 800 °C at a heating rate of 10 °C/min in a nitrogen gas environment.

## 3. Results and Discussion

### 3.1. Materials Parameters of GGBFS

**Figure 1:**
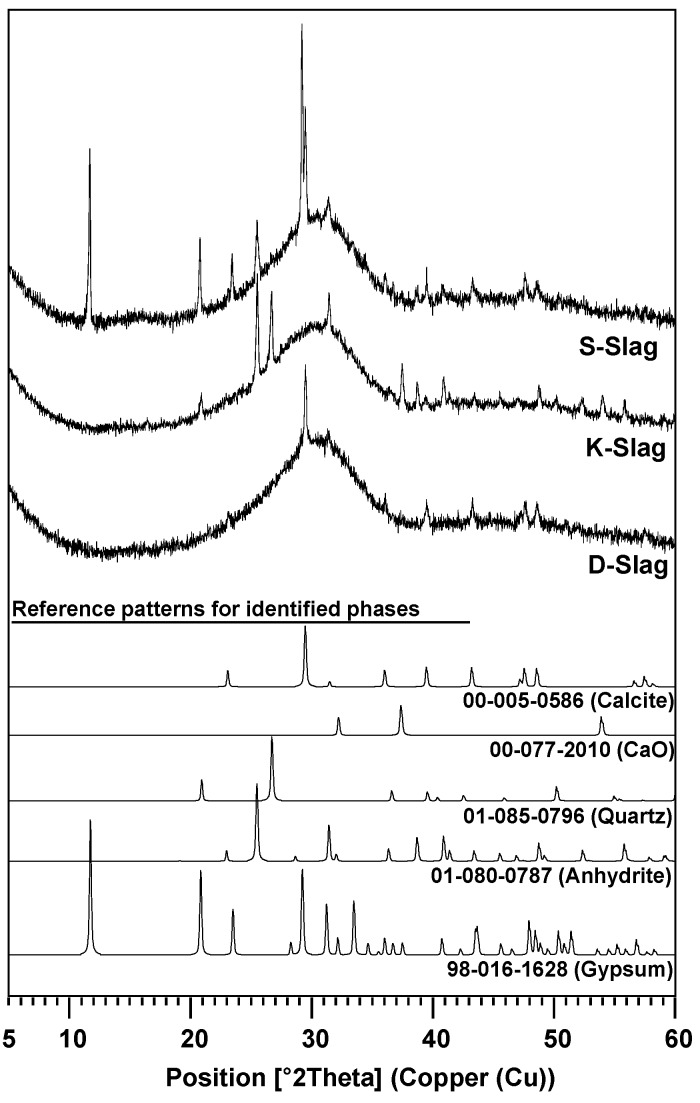
XRD patterns of raw ground granulated blast-furnace slag (GGBFS) samples with identified crystalline phases.

The XRD patterns of the raw GGBFSs are presented with identified phases in Figure 1. Mineralogical GGBFS compositions were semiquantitatively calculated using internal standard [12] and reference intensity ratio (RIR) methods [16]; the results are shown in Table 1.

Although quantifications using the RIR method are generally less accurate than those obtained via the Rietveld method, the RIR method was chosen for this study because (1) this method is much easier to use; and (2) the resolutions of the measured XRD patterns in this study were not sufficiently high to produce notable differences between these two methods. To increase the accuracy of the RIR method, more than 10 reference patterns for each phase were selected from the PDF-2 database and averaged for quantification.

In Table 1, anhydrite and gypsum are noted as the external chemicals, which are conventionally incorporated during the milling process for manufacturing GGBFS; CaO is often incorporated in Korea to meet the compositional requirements of the Korean Standards Association (KSA) for GGBFS (*i.e*., KSB > 1.6); thus, to correctly compare the content of the glass phase between GGBFSs, the anhydrite, gypsum, and CaO content should be excluded. After these chemicals were excluded, only small differences were found in the content of the raw GGBFS glass phases; however, the lowest content of S-Slag’s glass phase might indicate the lowest potential for strength development, although the relationship between glass content and strength remains uncertain [17].

The oxide compositions of the raw GGBFSs are presented in Table 2. Because it is known that the content of CaO, Al_2_O_3_, SiO_2_, MgO, Na_2_O, and K_2_O influence the hydraulic reactivity of GGBFS in blended Portland cements and cementless GGBFS binders [17], this study calculated three compositional indicators: basicity (KSB) (= (CaO + MgO + Al_2_O_3_)/SiO_2_) from the Korean Standards Association (KSA) [18]; the chemical modulus (CM) (= (CaO + MgO)/SiO_2_) [19]; and the atomic percentage of network-modifying elements in the glass phase (Net-MD) (= atomic percentage of (Ca + Na + K + Mg)) [17], as shown in Table 1. In Net-MD, the externally added fractions of Ca were excluded. Since their higher values suggest the higher potential reactivity of GGBFS, these indicators implied that the hydraulic reactivity of the S-Slag would be the highest, while that of the K-Slag would be the lowest. The LOI values per the TGA were 2.0%, 2.5%, and 2.0% in weight for the S-, K-, and D-Slags, respectively.

**Figure 2:**
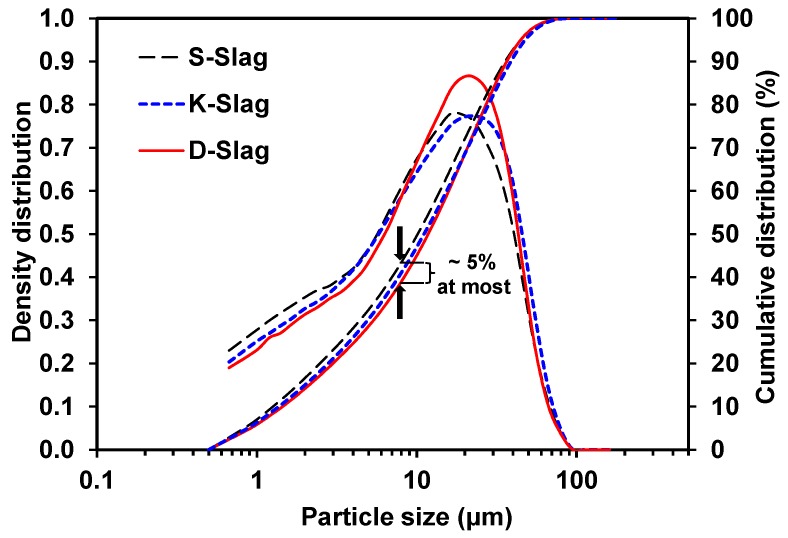
Particle-size distribution of raw GGBFS.

The particle-size distributions of raw GGBFS powders are presented in Figure 2. Overall, the particle-size distributions were relatively similar. As seen in Figure 2, S-Slag powder is the finest, while D-Slag powder is the coarsest, although the biggest difference between the cumulative curves appeared at a mere ~5%, around the size of 8 µm. Because the higher content of the small-sized particles tends to increase the hydraulic reactivity of GGBFS [20,21], S-Slag seems to be more favorable in terms of developing greater strength.

Table 3 summarizes all of the measured parameters, which are known to be important factors for producing higher hydraulic reactivity, with relative intensities from the lowest (●) to the highest (●●●) to compare the values of parameters between raw GGBFS samples. Overall, the S-Slag showed the highest potential, while the D-Slag demonstrated the lowest potential for high-strength production.

### 3.2. Characterization of Hardened Pastes

**Figure 3:**
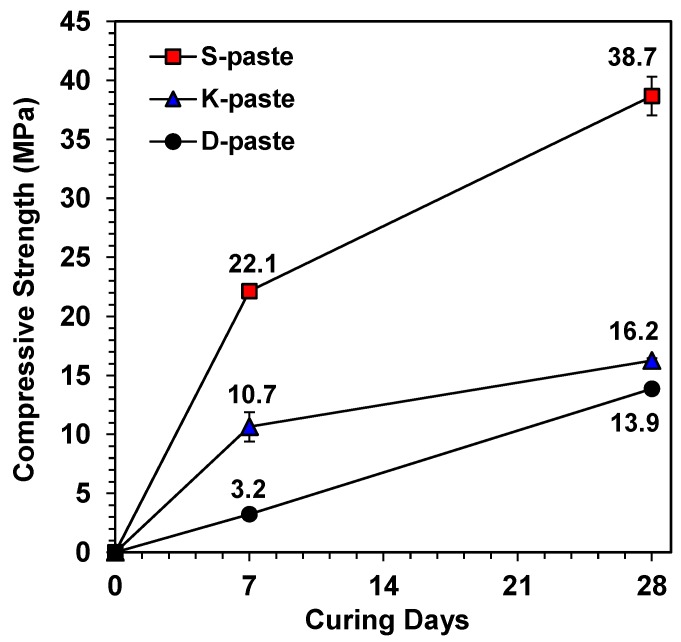
Strength developments of self-activation of GGBFS mixed with purified water.

The compressive strength testing results of all the hardened pastes are illustrated in Figure 3. The D-paste shows a curve similar to those that appear in the literature [5,6]; thus, the curve is probably typical of the self-activation of GGBFS without the addition of calcium sulfate for strength development; however, the S- and K-pastes demonstrate quite different strength developments. Although the greatest strength of the S-paste was expected, it was surprising that it was even greater than that of the Ca(OH)_2_- or NaOH-activated GGBFS described in earlier studies [7,22,23]. Note that the K- and D-pastes produced similarly low values of strength at 28 days; however, the early strength of the K-paste, at 7 days, was significantly higher than that of the D-paste; this is likely due to the presence of a sulfate source in K-Slag, because the calcium sulfate source could dramatically increase early strengths by forming ettringite [10].

In this study, the strength was not determined by any single dominant parameter; rather, it was determined by the comprehensive consequence of all the material parameters of GGBFS (Table 3). The relative comparison of these parameters predicted that the S- and D-pastes would produce the highest and the lowest strengths, respectively.

**Figure 4:**
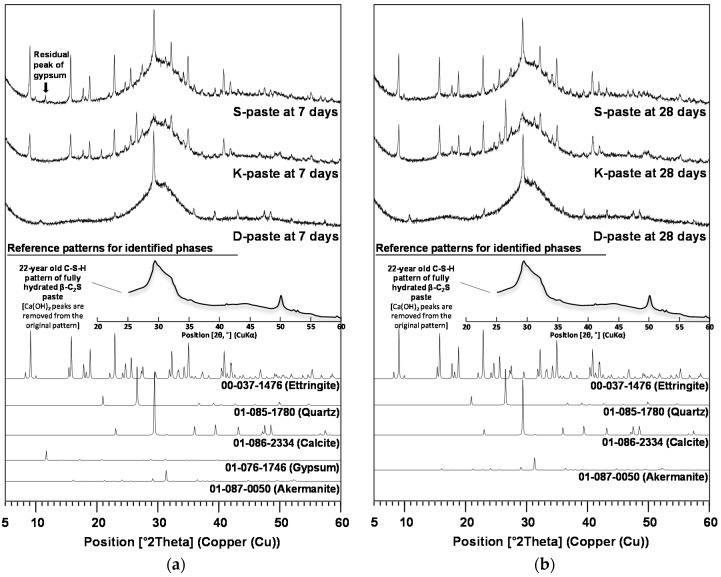
XRD patterns and identified phases for hardened pastes at (**a**) 7; and (**b**) 28 days.

Figure 4 shows the XRD patterns with the identified phases for hardened pastes at 7 and 28 days. In the XRD patterns, the reference patterns from the ICDD PDF-2 database were presented with the PDF numbers for the identified phases below the experimental XRD patterns; however, because the ICDD PDF-2 database did not contain any amorphous information, the reference pattern for C-S-H was taken from the XRD pattern of the 22-year-old β-C_2_S paste [24] after eliminating the peaks of Ca(OH)_2_.

As main reaction products, C-S-H (calcium silicate hydrate) appeared in all samples, but ettringite (Ca_6_Al_2_(SO_4_)_3_(OH)_12_·26H_2_O) was found only in the S- and K-pastes. While the S-paste showed few differences in the peak intensities of ettringite between 7 and 28 days, the K-paste peaks showed some growth from 7 to 28 days.

The XRD patterns suggest that very small quantities of akermanite (Ca_2_MgSi_2_O_7_) might be formed in all hardened pastes during hydration, since there was no akermanite in any of the raw GGBFS samples. The calcite (CaCO_3_) and quartz (SiO_2_) were the remaining crystalline phases from the raw GGBFS. Unlike previous studies on CaO- and Ca(OH)_2_-activated GGBFS [7,22], none of the samples showed any formation of Ca(OH)_2_ at any of the ages, probably due to a shortage of available Ca ions.

**Figure 5:**
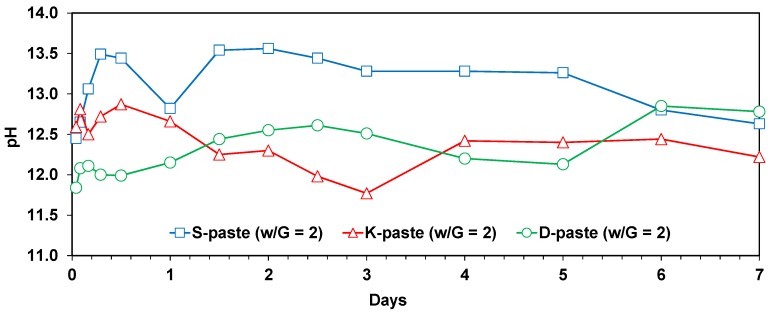
Temporal changes in pH values of diluted pastes of GGBFS powders (w/G = 2) for seven days.

The temporal changes of the pH values for the diluted pastes (w/G = 2) were monitored for seven days (as shown in Figure 5), during which the following observations were made: (1) The S-paste showed the highest pH values for most of the seven days; (2) the D-paste exhibited distinctively lower pH values (~12) than those of the K-paste (~12.7) during the first day; (3) however, the pH values of the D- and K-pastes became roughly similar after one day, although the values fluctuated over time.

An earlier study [4] reported that the pH values of pore solutions or diluted pastes were mainly governed by the dissolution of Ca, Na, and K ions from the GGBFS when it was mixed with pure water. When the pH increases, the dissolution of GGBFS is promoted [3,4,17]; thus, the higher pH value of the diluted paste indirectly indicates the higher degree of the dissolution of GGBFS in the paste.

In this study, the pH of diluted GGBFS pastes seemed to be more affected by (1) the particle-size distribution; (2) the alkali content; and (3) the calcium sulfate content, rather than the other material parameters such as glass content or chemical modulus [15]. As for the particle-size distribution, GGBFS with a higher fraction of small particles tended to show the higher pH value of the solution.

Despite the small alkali content (less than 1.1%) in all the GGBFS samples, the alkalis in raw GGBFS may increase the pH value of the diluted pastes more significantly than CaO or MgO [25]; note that a high pH value (~14) was often achieved by the dissolved alkalis in the pore solution of cementitious materials [3]. However, in this study, the alkali content in the GGBFS varied slightly, with differences that ranged from 0.6% to 1.1%. Therefore, rather than the alkali content, the additional calcium sulfate content (*i.e*., gypsum and anhydrite) could be more responsible for the pH, given that a high concentration of SO_3_ tends to increase the solubility of alkalis in cementitious materials [26].

The high content of alkalis and calcium sulfates in the K-Slag explains why the pH values of the K-paste were higher than those of the D-paste during the first 24 h; after 24 h, however, the extensive formation of ettringite in the K-paste (see Figure 4) may have led to the low pH values (~11.8) for the first three days due to the depletion of dissolved Ca and sulfate ions; after four days, the pH of the K-paste gradually increased due to the further dissolution of Ca; however, because there were few sulfate ions left in the K-paste, the dissolution behavior of K-Slag was likely to be similar to that of D-Slag.

Although the alkali content of S-Slag was the same as that of D-Slag, or even less than that of K-Slag, the significantly higher calcium sulfate content in S-Slag was likely to significantly increase the dissolution of alkalis, resulting in the highest pH values.

**Figure 6:**
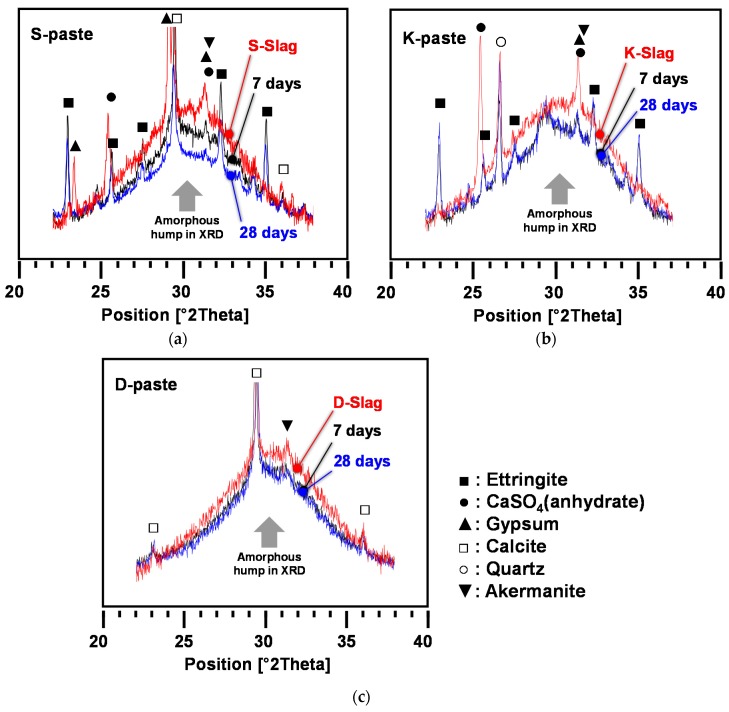
Gradual reduction of GGBFS glass phase with curing days. (**a**) S-paste; (**b**) K-paste; (**c**) D-paste.

Figure 6 illustrates the gradual reduction of the GGBFS glass phases, which were manifested in amorphous-hump changes, with curing days. Note that the continuing decrease of amorphous humps indicates the progressive dissolution of the GGBFS glass phase. The S-paste exhibited significant further dissolution of the glass phase beyond seven days, which must be responsible for the significant strength increase of the S-paste after seven days, while the K- and D-pastes showed much smaller amorphous-hump decreases before seven days and no further reduction after seven days.

The larger amorphous-hump reduction of the S-paste may be explained by the above discussions on pH and by several beneficial characteristics of S-Slag in terms of hydraulic reactivity, which are summarized in Table 3. In particular, according to the network theory by Zachariasen [17,27], Na, K, Ca, and Mg are the typical network-modifying elements in vitreous blast-furnace slag, and thus the highest content of these metallic elements of S-Slag was likely to increase the degree of depolymerization of the network structure of the amorphous phase, contributing to the higher degree of dissolution of glass in the S-paste.

The continuing dissolution of the glass phase after seven days in the S-paste was probably related to the residual gypsum at seven days. In this study, calcium sulfates were consumed mainly for producing ettringite. Despite the large formation of ettringite in the S-paste at seven days, unlike the K-paste, its XRD pattern continued to show residual amounts of gypsum. Given that the XRD analysis of the S-paste showed little change in terms of ettringite after seven days, the residual gypsum was probably used mainly for facilitating the dissolution of alkalis and the GGBFS glass phase beyond seven days.

**Figure 7:**
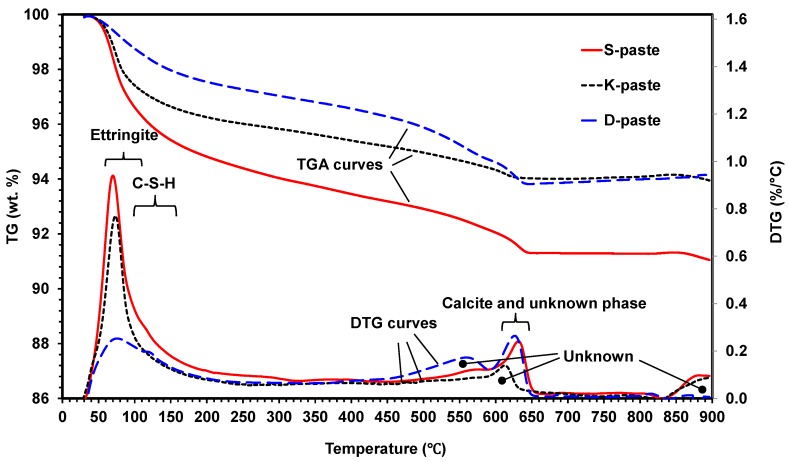
TGA and DTG results of 28-day hardened paste samples.

Figure 7 provides the TGA results with derivative of thermogravimetry (DTG) of the 28-day hardened samples with the identified phases. The total weight losses of all samples in this study appeared to be significantly smaller than those of the hardened GGBFS samples with various activators described in the literature [7,23]. The DTG results displayed C-S-H, ettringite, and calcite as major weight losses (see Table 4), and also showed a few unknown phases that were not identified in the XRD at several high temperatures over 500 °C. The D-paste did not show any formation of ettringite.

Note that all pastes similarly had peaks in the temperature range of calcite decomposition, 600–650 °C, in the DTG. However, the XRD results show that the weight loss in the K-paste was not a calcite but rather an unknown phase, because calcite was not detected in the XRD. Additionally, there was no possibility of calcite formation during the TGA test because the TGA test was performed in a nitrogen gas environment. Thus, the weight losses in the S- and D-pastes in that temperature range were likely to include not only calcite, but also the same unknown phase found in the K-paste.

It is difficult to accurately estimate the weight percentage of ettringite for hardened samples because its weight-loss peak closely overlaps with the C-S-H peak; however, note that the ettringite started to decompose at a temperature (near 80 °C) lower than that of the C-S-H (over 100 °C) [31]; thus, the greatest weight loss of the S-paste under 100 °C in the TGA indicates that it produced the largest quantity of ettringite, similar to the XRD result.

Up to a temperature of 200 °C, the S-paste displayed a much larger weight loss (*i.e*., C-S-H + ettringite) than that of the K-paste; thus, the large formation of ettringite and C-S-H could be one of the possible causes of the S-paste’s best strength.

Despite the large formation of ettringite in K-paste, it is interesting that the 28-day strength of the K-paste was only slightly greater than that of the D-paste. The D-paste may have produced a higher quantity of C-S-H than K-paste because the values of the DTG curves over 100 °C were similar between the D- and K-pastes, although the curve of the K-paste also contained a large ettringite peak.

## 4. Conclusions

This study collected three commercial GGBFS powders from Singapore, Korea, and the United Arab Emirates. These GGBFS samples were hydrated only with purified DI water to estimate their cementing capabilities as independent cementitious binders without activators. The following conclusions were made based on an analysis of the results of particle-size distribution, XRF, XRD, compressive strength tests, TGA, and pH measurement:

(1) The raw GGBFS samples showed quantitative and qualitative differences in the material parameters, which were compositional indicators (CM, KSB, and Net-MD), and particle-size distribution, as well as calcium-sulfate, alkali, and glass-phase content.
(2) In this study, the strength was not governed by any single dominant parameter, but was rather the comprehensive consequence of all material parameters of the raw GGBFS (summarized in Table 3); the relative comparison of these parameters predicted that the S- and D-pastes would produce the highest and the lowest strengths, respectively.
(3) S-Slag produced a high strength that was comparable to that of alkali- or Ca(OH)_2_-activated GGBFS.
(4) All paste samples produced C-S-H, but only the S- and K-pastes formed ettringite as a main-reaction product; the ettringite formation improved the early strength of the S- and K-pastes.
(5) The S-paste formed the largest amount of C-S-H and ettringite, which could be one of the possible causes for the best strength of the S-paste.
(6) The degree of dissolution of GGBFS with purified water was indirectly estimated through the monitoring of the pH of diluted GGBFS paste and the gradual reduction of amorphous humps in the XRD analysis, which were increased more by the smaller particle size, the higher alkali content, and the higher calcium-sulfate content of the GGBFS than by the other material parameters, such as a high glass content; in particular, the calcium-sulfate content appeared to be the most responsible for the dissolution.
(7) The highest degree of dissolution of GGBFS was found in the S-paste, which also showed the extended dissolution of the glass phase after seven days, unlike the other pastes. These also explain the best strength of the S-paste.

## Figures and Tables

**Table 1:** Mineralogical compositions of raw GGBFSs (wt.%) from XRD analysis.

Raw GGBFS	CaSO_4_ (Anhydrite)	CaSO_4_·2H_2_O (Gypsum)	CaO	Calcite	Quartz	Glass Phase	Glass Content Without Anhydrite and Gypsum
S-Slag	4.7	3.2	-	2.6	-	89.5	97.2
K-Slag	6.0	-	0.2	-	1.7	92.1	98.2
D-Slag	-	-	-	2.4	-	97.6	97.6

**Table 2:** Chemical compositions of raw GGBFSs from XRF analysis.

GGBFS	Oxide Composition (wt. %)										Parameters		
	CaO	SiO_2_	Al_2_O_3_	MgO	SO_3_	TiO_2_	K_2_O	Fe_2_O_3_	MnO	Na_2_O	KSB ^1^	CM ^2^	Net-MD ^3^
S-Slag	46.6	29.8	12.6	4.7	4.3	0.6	0.4	0.3	0.2	0.2	2.1	1.7	52.8
K-Slag	43.4	32.8	13.6	2.6	4.1	0.9	0.6	1.0	0.3	0.5	1.8	1.4	49.9
D-Slag	46.3	31.8	13.5	4.7	1.8	0.6	0.4	0.5	0.2	0.2	2.0	1.6	52.8

^1^ KSB (basicity) = (CaO + MgO + Al_2_O_3_)/SiO_2_ in weight ratio; ^2^ CM (chemical modulus) = (CaO + MgO)/SiO_2_ in weight ratio; ^3^ Net-MD (atomic % of network modifying elements in glass phase) = Ca + Na + K + Mg.

**Table 3:** Relative comparison of material parameters between raw GGBFS samples.

Positive Parameters for Increasing Hydraulic Reactivity of GGBFS	S-Slag	K-Slag	D-Slag
Values of CM and KSB	●●●	●●	●●●
Atomic % content of network modifying elements (Ca + Na + K + Mg) (Net-MD) in glass phase	●●●	●●	●●●
Content of calcium sulfate source	●●●	●●	●
Fraction of smaller particles	●●●	●●	●
Content of alkalis (*i.e*., Na_2_O and K_2_O)	●●	●●●	●●
Content of glass phase without external chemicals	●●	●●●	●●

* ●●●: relatively high; ●●: relatively intermediate; ●: relatively low.

**Table 4:** Temperature range of weight loss for reaction products from TGA analysis.

Phase	Temperature Range of Weight Loss
C-S-H	105 °C [28] 120–145 °C [25] 106 ± 4 °C for first peak, 129 ± 4 °C for second peak [29] C-S-H loses its water over a broad temperature range, but the maximum loss occurs at 120–160 °C [30] 100–200 °C for C-S-H(I), 115–125 °C for C-S-H [3]
Ettringite	183 ± 3 °C [29] 80–130 °C [30,31]
Gypsum	80–220 °C (max. 167 °C) via transformation to anhydrite [32] 100–200 °C due to dehydration [33]
Calcite (CaCO_3_)	720–760 °C [25] 680–760 °C [30]
